# Supplementary material for: Consequences of measurement error in qPCR telomere data: A simulation study
Source: PLoS One. 2019 May 1;14(5):e0216118. doi: 10.1371/journal.pone.0216118 (PMC6493763; doi:10.1371/journal.pone.0216118)
Supplement: S1 File — (PDF) [file pone.0216118.s001.pdf]

Supporting Information for Nettle et al. 'Consequences of measurement error in qPCR telomere data: A simulation study'

Section 1: Analytical treatment of measurement error in the TS ratio

This section examines the TS ratio under measurement error, providing analytical results to support simulation findings.

As specified in the main paper, the ideal (i.e. error-free) Cq values for the telomere assay and single-copy gene relate to the amounts of each kind of DNA present in the sample as given in (1) and (2).

$$iCq_s = f - \log(DNA_s) \quad (1)$$

$$iCq_t = f - \log(DNA_t) \quad (2)$$

Here, f denotes a constant set by the chosen fluorescence threshold. The amount of telomeric DNA present is proportional to the amount of single-copy gene DNA present, but scaled by the relative telomere length of the individual.

$$DNA_t = a \cdot tl \cdot DNA_s \quad (3)$$

Hence, combining equations (2) and (3):

$$iCq_t = f - \log(a \cdot tl \cdot DNA_s) \quad (4)$$

The measured Cq values are the true Cq values plus a measurement error term, as follows:

$$mCq_s = f - \log(DNA_s) + \varepsilon_s \quad (5)$$

$$mCq_t = f - \log(a \cdot tl \cdot DNA_s) + \varepsilon_t \quad (6)$$

Here, $\varepsilon_t \sim N(0, \sigma_{\varepsilon t})$ and $\varepsilon_s \sim N(0, \sigma_{\varepsilon s})$. The formulae for the ideal (i.e. error-free) and measured TS ratio are given in (7) and (8).

$$iTS = 2^{-(iCq_t - iCq_s)} \quad (7)$$

$$mTS = 2^{-(mCq_t - mCq_s)} \quad (8)$$

Reference Cq values for a standard sample are typically subtracted from the Cqs for the single-copy gene and telomeric assay when calculating TS ratios. The effect of this is simply to rescale the TS ratio; such rescaling can be ignored in what follows without loss of generality, and hence for clarity we do not include this step here (though see main paper for the TS formula with these reference values included).

By substituting into (7) and (8) and rearranging, we have:

$$iTS = 2^{-(f - \log(a \cdot tl \cdot DNA_s) - f + \log(DNA_s))}$$

$$iTS = 2^{(\log(a \cdot tl \cdot DNA_s) - \log(DNA_s))}$$

$$iTS = 2^{(\log\left(\frac{a \cdot tl \cdot DNA_s}{DNA_s}\right))}$$

$$iTS = a \cdot tl \quad (9)$$

Thus, (9) gives us **Result 1:** The TS ratio, if measured without error, is proportional to the relative telomere length in the sample.

For the measured TS ratio where there is measurement error, we have:

$$mTS = 2^{-(f - \log(a \cdot tl \cdot DNA_s) + \varepsilon_t - f + \log(DNA_s) - \varepsilon_s)}$$

$$mTS = 2^{(\log(a \cdot tl \cdot DNA_s) - \varepsilon_t - \log(DNA_s) + \varepsilon_s)}$$

$$mTS = 2^{\left(\log\left(\frac{a \cdot tl \cdot DNA_s}{DNA_s}\right) - \varepsilon_t + \varepsilon_s\right)}$$

$$mTS = 2^{\left(\log\left(\frac{a \cdot tl \cdot DNA_s}{DNA_s}\right) - \varepsilon_t + \varepsilon_s\right)}$$

$$mTS = 2^{(\varepsilon_s - \varepsilon_t)} a \cdot tl \qquad (10)$$

From (10), we have **Result 2:** The measured TS ratio is proportional to relative telomere length multiplied by $2^{(\varepsilon_s - \varepsilon_t)}$, or two to the power of the difference between the measurement errors in the two Cq values.

The error in the measured TS ratio (henceforth ε_{TS}) is the difference between the measured TS ratio, mTS, and the ideal or error-free TS ratio, iTS. From (9) and (10):

$$\varepsilon_{TS} = 2^{(\varepsilon_s - \varepsilon_t)} a \cdot tl - a \cdot tl$$

$$\varepsilon_{TS} = \left(2^{(\varepsilon_s - \varepsilon_t)} - 1\right) a \cdot tl \qquad (11)$$

By inspection of (11), we have **Result 3:** The error in the TS ratio is proportional to telomere length. This is true even though the errors in the Cq values were assumed to be independent of the amounts of telomere and single-copy DNA in the samples.

If $\varepsilon_t \sim N(0, \sigma_{\varepsilon t})$ and $\varepsilon_s \sim N(0, \sigma_{\varepsilon s})$, from properties of the normal distribution:

$$\varepsilon_s - \varepsilon_t \sim N\left(0, \sqrt{\sigma_{\varepsilon s}^2 + \sigma_{\varepsilon t}^2 - 2\rho\sigma_{\varepsilon s}\sigma_{\varepsilon t}}\right)$$

Here, ρ is the correlation between ε_s and ε_t. Hence, the distribution of ε_{TS} is the distribution of:

$$\left(2^{N\left(0, \sqrt{\sigma_{\varepsilon s}^2 + \sigma_{\varepsilon t}^2 - 2\rho\sigma_{\varepsilon s}\sigma_{\varepsilon t}}\right)} - 1\right) a \cdot tl \qquad (12)$$

From (12), we can make the following inferences for the case where the measurement errors in the Cq values are normally distributed:

- **Result 4:** Positive correlations between ε_s and ε_t reduce the size of measurement errors in the TS ratio. From (12), given that $2\sigma_{\varepsilon t}\sigma_{\varepsilon s}$ is positive, increasing ρ will always reduce the size of $\sqrt{\sigma_{\varepsilon s}^2 + \sigma_{\varepsilon t}^2 - 2\rho\sigma_{\varepsilon s}\sigma_{\varepsilon t}}$, and hence the standard deviation of ε_{TS}.
- **Result 5:** Perfect positive correlation between the measurement errors of the Cq for telomere and the Cq for the single-copy gene eliminates measurement error in the TS ratio entirely, as long as the extent of measurement error is the same for the two reactions. Where $\rho = 1$ and $\sigma_{\varepsilon t}^2 = \sigma_{\varepsilon s}^2$, $\sqrt{\sigma_{\varepsilon s}^2 + \sigma_{\varepsilon t}^2 - 2\rho\sigma_{\varepsilon s}\sigma_{\varepsilon}} = 0$. Hence, from (12), the measurement errors in the TS ratio are: $\left(2^{N(0,0)} - 1\right) a \cdot tl = 0$.

If we can assume that telomere length itself is normally distributed, then we can see from (12) that the error in the TS ratio contains a normally distributed component (*tl*) and a log-normally

distributed component (since the logarithm of $2^{N\left(0,\sqrt{\sigma_{\varepsilon s}^2+\sigma_{\varepsilon t}^2-2\rho\sigma_{\varepsilon s}\sigma_{\varepsilon t}}\right)}$ is by definition normally distributed, $2^{N\left(0,\sqrt{\sigma_{\varepsilon s}^2+\sigma_{\varepsilon t}^2-2\rho\sigma_{\varepsilon s}\sigma_{\varepsilon t}}\right)}$ is log-normal). Thus, the distribution of ε_{TS} belongs to the class of normal-log-normal mixture distributions. Such distributions are typically skewed and leptokurtic (Yang 2008).

Section 2: Simulation results with correlations between errors

Simulation results reported in the main paper assume that the error in the telomere Cq and the error in the single-copy gene Cq are uncorrelated; that is, in the notation of section 1, $\rho = 0$. We repeated the main simulations assuming positive values of ρ. Increasing values of ρ attenuate the impact of measurement error at the Cq level on the TS ratio (see section 1, result 5). Although $\rho = 1$ makes the TS ratio error-free regardless of the magnitude of error in Cqs (see section 1, result 6), the effect of more modest non-zero values of ρ is slight. For example, Fig S1 shows how repeatability of *mTS* relates to the error σ values under three different assumptions about ρ, namely zero correlation (repeating Fig 4A of the main paper), a weak correlation, and a strong correlation. Even assuming a strong correlation, error σ values of less than around 0.15 are still necessary for repeatabilities above 0.6.

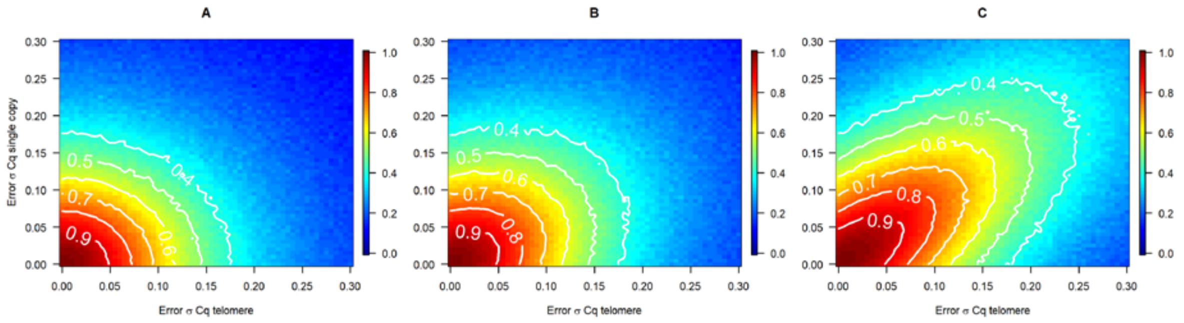

Fig S1. Repeatability of the TS ratio (intra-class correlation coefficient) as the error σ values for the telomere assay and the single-copy gene vary. A: Errors are uncorrelated. B: Weak positive correlation ($\rho = 0.3$) between the errors. C: Strong positive correlation ($\rho = 0.7$) between the errors. Simulations of n=10000 are used at each 0.005 step of error σ, with other parameters having their default values.

Repeating other simulated results with positive values of ρ produces similar conclusions: increasing ρ attenuates the impact of error in measuring Cqs on the TS ratio, but the effect is slight until ρ is close to 1.

Section 3: How to use the simulation R code

We define a series of R functions, contained in the script 'simulation.functions.r', that return datasets with requested properties containing both the true values of the quantities (Cqs, TS, etc.), and their post-error measured values. This allows the user to determine the differences between true and measured values, and perform other analyses. All simulation parameter values are user-specifiable. The script 'paper.results.r' reproduces all the figures and simulation results from the main paper.

Datasets consist of observations from *n* individuals. The steps common to all of the simulation functions are as follows:

- A vector of *n* true single-copy gene abundances, *true.dna.scg* is defined, drawn from a normal distribution with mean *b* and standard deviation *var.sample.size* (*b* is a constant).
- A vector of *n* relative telomere lengths, *true.telo.var* is defined, drawn from a normal distribution with mean 1 and standard deviation *telomere.var.*
- Hence, the true abundance of the telomere sequence is defined, as *a*true.dna.scg*true.telo.var*. Here, *a* is a scaling constant representing how many copies of the telomeric sequence there are per single-copy gene in the average sample.
- Ideal Cq values for both reactions are defined as $f - log_2(true.dna.scg)$ and $f - log_2(true.dna.telo),$ where *f* is a constant representing the chosen fluorescence threshold.
- Measurement errors in the Cqs are generated from a normal distribution with mean 0; standard deviations given by *error.scg* and *error.telo*; and a correlation between *error.scg* and *error.telo* given by *error.cor*.
- Hence, measured Cqs are generated, which can be compared to the ideal Cq values.
- TS ratios are calculated both on the measured Cqs, and the ideal ones.

The following functions are available. Specify desired parameter values in the parenthesis, e.g. *generate.one.dataset(n=10000, error.telo=0.1, error.scg=0.1, error.cor=0*). Default values in the simulation functions are generally those given in table 1 of the main paper.

- *generate.one.dataset()* returns a simple dataset (one telomere measurement per individual) for chosen values of all the variables described in section 1. As well as ideal and measured Cqs, it returns ideal and measured TS ratios. It also returns the difference between the ideal and measured TS ratio, calculated two ways, computed (*error.computed*), and using equation (11) of online supplement 1 (*error.analytic*). Both methods produce the same number. This was included as an additional check of correctness of the simulation.
- *generate.repeated.measure()* returns a dataset where telomere lengths from the same individuals are measured twice, via two independent biological samples, and the true telomere length of each individual is assumed not to have changed at all. The data frame it returns is as for *generate.one.dataset()*, except that there are two of each variable (e.g. *true.ts.1, true.ts.2, measured.ts.1, measured.ts.2*, etc.).
- *calculate.repeatability()* calculates the repeatability of the measured TS ratio (intra-class correlation coefficient) when *generate.repeated.measure()* is implemented using the given values for all the parameters. It requires prior installation of R package 'irr'.
- *compare.repeatability()* returns the repeatability of the TS ratio and the repeatability calculated on the raw Cqs for the telomere reaction, for the given parameter values.
